# Trifluoromethyl ethers – synthesis and properties of an unusual substituent

**DOI:** 10.3762/bjoc.4.13

**Published:** 2008-04-29

**Authors:** Frédéric R Leroux, Baptiste Manteau, Jean-Pierre Vors, Sergiy Pazenok

**Affiliations:** 1 Laboratoire de Stéréochimie, Université Louis Pasteur (ECPM), CNRS, 25 rue Becquerel, F – 67087 Strasbourg Cedex 2, France; 2 Bayer CropScience SA, Centre de Recherches de La Dargoire, 14-20 Rue Pierre Baizet, F – 69009 Lyon, France; 3 Bayer CropScience AG, Alfred-Nobel-Strasse 50, D – 40789 Monheim, Germany

## Abstract

After nitrogen, fluorine is probably the next most favorite hetero-atom for incorporation into small molecules in life science-oriented research. This review focuses on a particular fluorinated substituent, the trifluoromethoxy group, which is finding increased utility as a substituent in bioactives, but it is still perhaps the least well understood fluorine substituent in currency. The present review will give an overview of the synthesis, properties and reactivity of this important substituent.

## Introduction

Nowadays, fluorine containing compounds are synthesized in pharmaceutical research on a routine basis and about 10% of all marketed pharmaceuticals contain a fluorine atom. There has been an enormous increase in the use of fluorine containing compounds for medicinal applications. For example, nine of the 31 new chemical entities approved in 2002 contain one or several fluorine atoms. According to the World Drug Index (WDI), there are 128 fluorinated compounds with US trade names. Even more fluorinated drugs are predicted to be developed in the near future, as fluoro-organic compounds continue to attract attention in the field of chemistry and biochemistry [1].

**Figure 1:**
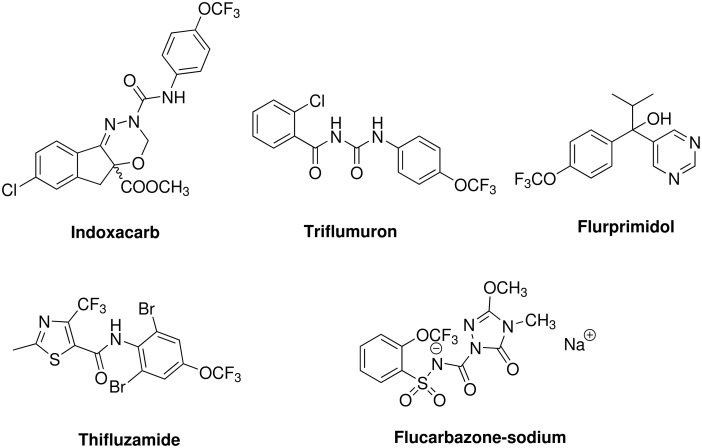
OCF_3_-bearing pesticides.

Fluorine as a substituent in active ingredients plays a significant and increasingly important role. Currently about 15% of the pesticides listed in the 13th edition of the Pesticide Manual contain at least one fluorine atom. The biggest group of fluorinated pesticides are the compounds containing a trifluoromethyl group (mainly at aromatic rings), followed by aromatic compounds containing an isolated fluorine atom (one and more). However, according to the 12th and 13th edition of the pesticide manual only five pesticides containing OCF_3_-groups are so far registered (see Figure 1). The proinsecticide Indoxacarb acting as a voltage-gated sodium channel (vgSCh) modulator, the insect growth regulant (IGR) Triflumuron, the plant growth regulator Flurprimidol, the inhibitor of the respiratory chain and succinate dehydrogenase (SD) Thifluzamide as well as the inhibitor of acetolactate synthase (ALS) Flucarbazone-sodium. It was estimated that the number of fluorinated compounds currently under development represent some 35–50% of the all active ingredients under development [2].

One or several fluorine atoms as substituents at specific sites in an organic compound can dramatically alter its chemical and biological nature. In fact, the incorporation of fluorine into a bioactive compound allows a simultaneous change in the electronic, lipophilic and steric parameters, all of which can influence both the pharmacodynamic and pharmacokinetic properties of the candidate [3].

What is so particular about fluorine? Due to its comparable size, the fluorine atom (1.47 Å) can mimic a hydrogen atom (1.20 Å) or a hydroxy group (1.40 Å) in a bioactive compound with respect to steric requirements at receptor sites. Its high electronegativity (4.0 according to the Pauling scale) can have a pronounced influence on the reactivity pattern of a molecule. The most common reason for incorporating fluorine into a molecule is to reduce the rate of oxidative metabolism. However, the increased oxidative stability of fluorinated molecules has nothing to do with the greater strength of the carbon-fluorine bond relative to the carbon-hydrogen bond. In fact, biological oxidation does not involve the homolysis of C–H or C–F bonds. More relevant are the bond energies and heats of formation of H–O and C–O bonds relative to those of F–O bonds. As the latter are unfavorable all alternative mechanisms avoiding attack at fluorine always apply in biological systems [4].

Moreover, the presence of fluorine atoms in biologically active molecules can enhance their lipophilicity and thus their *in vivo* uptake and transport. In particular, the trifluoromethyl group (−CF_3_) confers increased stability and lipophilicity in addition to its high electronegativity [5–9]. However, another fluorinated substituent, the trifluoromethoxy group, is becoming more and more important in both agrochemical research and pharmaceutical chemistry [10–11].

The trifluoromethoxy group is perhaps the least well understood fluorine substituent. When asked to draw up a list of textbook substituents, hardly anyone would consider associating such an "exotic entity" like trifluoromethoxy to the lasting popularity of the carboxyl, acetyl, formyl, nitro, amino, hydroxyl and sulfo groups. Nevertheless, the occurrence of OCF_3_-substituted organics, the majority of which are aromatic compounds, has significantly increased in the recent years [12].

In the 1950s and 1960s the successful development of α-fluorinated ethers as volatile, non-toxic, non-explosive and fast-acting inhalation anesthetics was quickly followed by applications of anti-inflammatory agents. Investigations of the anesthetic properties of α-fluorinated ethers were undertaken on the rational basis that replacement of the hydrogen atom in already known "anesthetic molecules" by fluorine should result in derivatives having improved thermal stabilities relative to the inhalation anesthetics in common use at that time (cyclopropane and ether), like the halo ether anesthetic Fluoroxene (Fluoromar®, F_3_C-H_2_C-O-CH=CH_2_). Numerous analogues [13] were prepared and evaluated (Table 1). Meanwhile, cyclic analogues bearing the fluorinated 1,3-dioxolanes moiety [14] have largely replaced Fluoroxene in its clinical use. Many anesthetics currently used are powerful positive allosteric modulators of GABA_A_ [15].

**Table 1:** α-Fluorinated ethers used as Anesthetics.

Entry	α-Fluorinated ethers	b.p. [°C]	Common names	Brand names

1	F_2_HC-O-CHFCF_3_	12.4 ± 25.0	Desflurane	Suprane®
2	F_2_HC-O-CHClCF_3_	48.5 ± 0.0	Isoflurane	Forane®
3	FH_2_C-O-CH(CF_3_)_2_	49.5 ± 25.0	Sevoflurane	Sevofrane®
4	F_2_HC-O-CF_2_-CHFCl	59.9 ± 25.0	Enflurane	Ethrane®
5	F_2_HC-O-CHF-CF_2_-CHF_2_	60.9 ± 25.0	BAX 3224	Synthane®
6	H_3_C-O-CF_2_-CHFBr	87.0 ± 25.0	Roflurane	DA 893
7	H_3_C-O-CF_2_-CHCl_2_	105.0 ± 0.0	Methoxyflurane	Pentrane®

Numerous new OCF_3_ containing compounds have been prepared, clinically evaluated and in many cases marketed as drugs with enhanced effectiveness, often coupled with diminished side-effects [10]. Between 2004 and 2007 the number of structures bearing an OCF_3_-substituent has more than doubled (from 30,000 to 74,514). They are documented in 18,000 literature references (SciFinder Scholar), most being patent applications (~11,000), but also in close to 7000 research articles. In contrast, trifluoromethoxy substituted heterocycles are relatively rare, although numerous structures are protected by patent applications.

## Review

### Preparation of Trifluoromethyl Ethers

#### Nucleophilic substitution

**Scheme 1:**
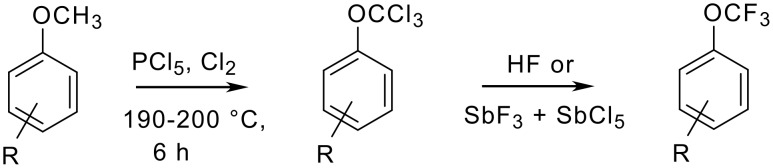
Preparation of trifluoromethyl ethers via a chlorination/fluorination sequence.

The first aryl trifluoromethylethers were prepared by L. Yagupol'skii in 1955 starting from substituted anisoles [16]. The displacement of chlorine by fluorine was realized with anhydrous hydrogen fluoride or with antimony trifluoride in the presence of antimony pentachloride (Scheme 1 and Table 2) [16–19].

**Table 2:** Synthesis of ArOCF_3_ compounds starting from substituted anisoles.

Anisole	ArOCCl_3_	Yield (%)	ArOCF_3_	Yield (%)

4-ClC_6_H_4_OMe	4-ClC_6_H_4_OCCl_3_	77	4-ClC_6_H_4_OCF_3_	80
2-ClC_6_H_4_OMe	2-ClC_6_H_4_OCCl_3_	69	2-ClC_6_H_4_OCF_3_	40
4-FC_6_H_4_OMe	4-FC_6_H_4_OCCl_3_	66	4-FC_6_H_4_OCF_3_	58
2,4-Cl_2_C_6_H_3_OMe	2,4-Cl_2_C_6_H_3_OCCl_3_	70	2,4-Cl_2_C_6_H_3_OCF_3_	20
4-NCC_6_H_4_OMe	4-NCC_6_H_4_OCCl_3_	50	4-NCC_6_H_4_OCF_3_	20
4-Cl(O)CC_6_H_4_OMe	4-Cl(O)CC_6_H_4_OCCl_3_	83	4-F(O)CC_6_H_4_OCF_3_	69

**Scheme 2:**
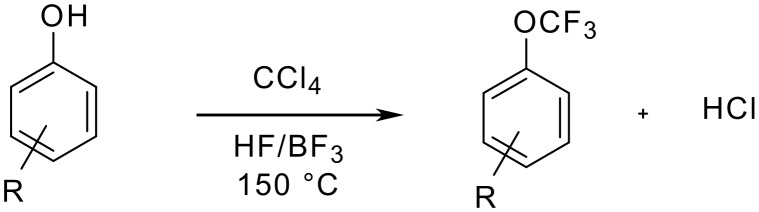
Preparation of trifluoromethyl ethers via an *in situ* chlorination/fluorination sequence.

The photochlorination which works well with electron-deficient anisoles cannot be applied to anisole itself. In fact, halogen attack on the phenyl ring proceeds more easily than radical chlorination of the methyl group. Louw and Franken could show that with elemental chlorine, photostimulated in refluxing tetrachloromethane, essentially trichloromethylanisole is obtained [20]. The fluorination of the trichloromethyl ether succeeds then easily as shown above. The chlorination/fluorination sequence described above can be simplified by producing the trichloromethyl aryl ethers without isolation and through *in situ* conversion into the final trifluoromethyl aryl ethers. As Feiring could show more recently, the phenol is heated together with tetrachloromethane, anhydrous hydrogen fluoride and catalytic amounts of boron trifluoride in a closed pressure vessel under autogeneous pressure up to 150 °C [21]. However, substrates containing *ortho* substituents capable of hydrogen bonding to the hydroxy group are not suitable starting materials. The stoechiometric use of tetrachloromethane lowers the yield and milder conditions afford essentially chlorodifluoromethoxy derivatives (Scheme 2 and Table 3).

**Table 3:** Synthesis of ArOCF_3_ compounds via an *in situ* Cl/F exchange.

Phenol (mol)	CCl_4_ (mol)	HF (g)	Conditions	ArOCF_3_	Yield (%)

C_6_H_5_OH			100 °C/2 h	C_6_H_5_OCF_3_	10
(0.05)	0.15	30	150 °C/4 h		
4-O_2_NC_6_H_4_OH			150 °C/8 h	4-O_2_NC_6_H_4_OCF_3_	56
(0.15)	0.15	40			
4-O_2_NC_6_H_4_OH			100 °C/8 h	4-O_2_NC_6_H_4_OCF_2_Cl	45
(0.06)	0.15	40			
4-ClC_6_H_4_OH			150 °C/8 h	2-ClC_6_H_4_OCF_3_	70
(0.6)	1.8	400			
3-H_2_NC_6_H_4_OH			140 °C/8 h	3-H_2_NC_6_H_4_OCF_3_	26
(0.6)	1.8	400			
2-FC_6_H_3_OH			150 °C/8 h	2-FC_6_H_3_OCF_3_	35
(0.07)	0.21	40			
4-MeC_6_H_4_OH			100 °C/2 h	4-MeC_6_H_4_OCF_3_	20
(0.05)	0.12	30	150 °C/4 h		

**Scheme 3:**
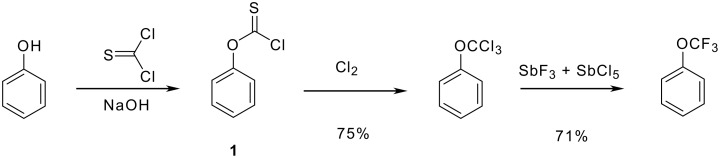
Preparation of trifluoromethyl ethers via chlorothionoformates.

Yarovenko and Vasil'eva developed an approach based on the readily accessible, although highly toxic aryl chlorothionoformates **1**. They can be cleanly converted by chlorination into trichloromethyl aryl ethers [17]. This step is then followed by fluorination using antimony trifluoride and a catalytic amount of antimony pentachloride (Scheme 3). The latter compounds can be obtained directly when treated with molybdenum hexafluoride [22]. Unfortunately, the high percutaneous toxicity of the chlorothionoformates **1** prohibited any industrial exploitation so far.

**Scheme 4:**
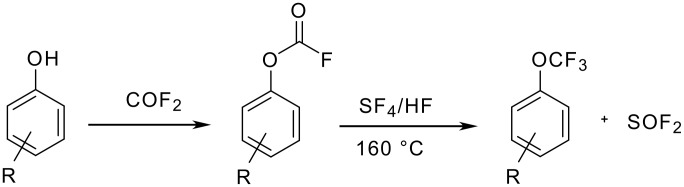
Preparation of trifluoromethyl ethers via fluoroformates.

W. Sheppard described in 1964 the syntheses of aryl trifluoromethylethers [23] by reaction of SF_4_ with aryl fluoroformates. However, this approach implied the use of highly toxic reagents and the fluoroformates were rarely isolated (Scheme 4 and Table 4).

**Table 4:** Preparation of aryl trifluoromethyl ethers by two-step method from phenols*^a^*.

Phenol (mol)	COF_2_ (mol)	SF_4_ (mol)	ArOCF_3_	Yield (%)

C_6_H_5_OH			C_6_H_5_OCF_3_	62
(2.5)	3.0	2.5		
4-O_2_NC_6_H_4_OH			4-O_2_NC_6_H_4_OCF_3_	81
(1.0)	1.5	1.5		
4-ClC_6_H_4_OH			2-ClC_6_H_4_OCF_3_	58
(0.25)	0.38	0.28		
2-ClC_6_H_4_OH			2-ClC_6_H_4_OCF_3_	17
(0.25)	0.38	0.28		
4-FC_6_H_4_OH			4-FC_6_H_4_OCF_3_	42
(0.13)	0.22	0.16		
4-MeC_6_H_4_OH			4-MeC_6_H_4_OCF_3_	9
(1.0)	1.35	1.2		

*^a^*Reactions run in "Haselloy-lined" pressure vessel of 140, 240, or 1000 mL. capacity at autogenous pressure. Normal heating pattern was 1 h at 100 °C followed by 2 to 3 h at 140 °C (or higher temperatures above phenol melting point) for the COF_2_ reaction; 2 h successively at 100, 140, or 150 °C, and 160 or 175 °C for the SF_4_ reaction.

#### Fluorodesulfurization methods

**Scheme 5:**
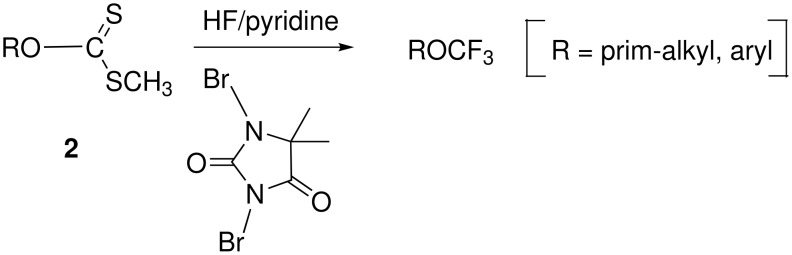
Oxidative desulfurization-fluorination toward trifluoromethyl ethers.

Recently, an elegant method towards trifluoromethyl ethers based on an oxidative desulfurization-fluorination has been disclosed by Hiyama [24–27]. When dithiocarbonates (**2**, xanthogenates) are exposed to a huge excess of hydrogen fluoride-pyridine and 1,3-dibromo-5,5-dimethylhydantoin, trifluoromethyl ethers form in moderate to excellent yields (Scheme 5 and Table 5).

**Table 5:** Oxidative desulfurization-fluorination towards ROCF_3_ compounds.

Xanthogenate **2**	Fluoride source*^a^* (mol)	*N*-halo imide*^b^* (mol)	ArOCF_3_*^c^*	Yield (%)

4-*n*-Pr-C_6_H_4_-	70% HF/Py (40)	DBH (3)	4-*n*-Pr-C_6_H_4_-OCF_3_	81
	TBAH_2_F_3_ (5)	NBS (4)	4-*n*-Pr-C_6_H_4_-OCF_2_SMe	58
4-*n*-Hex-C_6_H_4_-	70% HF/Py (80)	DBH (3)	4-*n*-Hex-C_6_H_4_-OCF_3_	50
4-PhCH_2_O-C_6_H_4_-	70% HF/Py (80)	DBH (4)	4-PhCH_2_O-C_6_H_4_-OCF_3_	56
4-Br-C_6_H_4_-	70% HF/Py (80)	DBH (3)	4-Br-C_6_H_4_-OCF_3_	62
Ph-CH_2_CH_2_CH_2_-	70% HF/Py (80)	DBH (3)	Ph-CH_2_CH_2_CH_2_-OCF_3_	75
*n*-C_16_H_33_-	70% HF/Py (80)	DBH (3)	*n*-C_16_H_33_-OCF_3_	95

*^a^*Mol amounts of HF/Py (70%) or tetrabutylammonium dihydrogen trifluoride (TBAH_2_F_3_) for 1 mol of **2**. *^b^*Mol amounts of 1,3-dibromo-5,5-dimethylhydantoin (DBH) or *N*-bromosuccinimide (NBS). *^c^*Isolated yield.

**Scheme 6:**
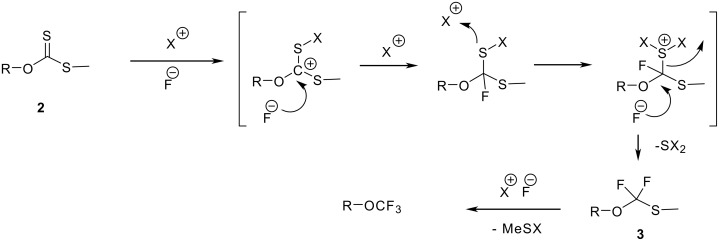
Mechanism of the oxidative desulfurization-fluorination.

What makes this procedure attractive is its applicability to the conversion of aliphatic alcohols into trifluoromethyl alkyl ethers provided that the alcohol is primary rather than benzylic, secondary or tertiary (in which case the reaction fails). The mechanism is based on the nucleophilic attack of the carbon-sulfur bond on a positively charged halogen which makes subsequently the nucleophilic substitution by a fluoride possible (Scheme 6). Under modified reaction conditions, for example by using TBAH_2_F_3_ instead of HF-pyridine, the transient monothioacetals **3** can be isolated [24].

#### CF_3_-Transfer agents

**Scheme 7:**
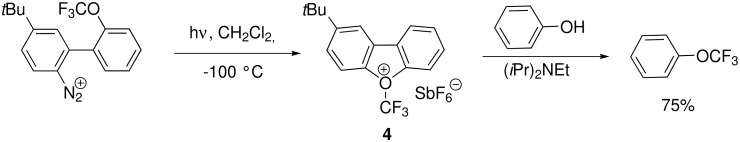
Umemoto's *O*-(trifluoromethyl)dibenzofuranium salts **4** as CF_3_-transfer agents.

Umemoto reported recently in detail on the preparation of *O*-(trifluoromethyl)dibenzofuranium salts **4** [28–31] and their use as CF_3_-transfer agents based on studies of Yagupol'skii [32]. The direct *O*- and *N*-trifluoromethylation of alcohols, phenols, amines, anilines and pyridines under mild conditions was described. However, the difficulty in the use of these reagents is the *in situ* preparation by photochemical decomposition of the corresponding 2-(trifluoromethoxy)biphenylyl-2'-diazonium salts at −100 °C (Scheme 7) [28]. The major drawback of this method is the necessity to work at very low temperature and on small scale.

**Scheme 8:**
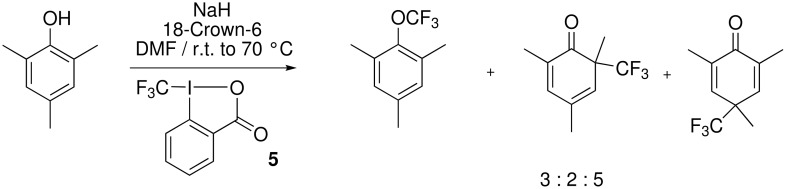
Togni's approach using hypervalent iodine compounds as CF_3_-transfer agents.

Togni managed very recently to circumvent these difficulties by using hypervalent iodine compounds such as **5** [33–35]. When 2,4,6-trimethylphenol was treated with the hypervalent iodine compound depicted below, the corresponding trifluoromethyl ether was obtained beside C-trifluoromethylation products (Scheme 8).

Alkyl trifluoromethyl ethers, still a rarity, have so far been prepared by the reaction of alkyl fluoroformates with sulfur tetrafluoride [36], the trifluoromethyl transfer from *O*-(trifluoromethyl)dibenzofuranium hexafluoroantimonate **4** [37] and the addition of trifluoromethyl hypofluorite (FOCF_3_) to olefins [38].

**Scheme 9:**
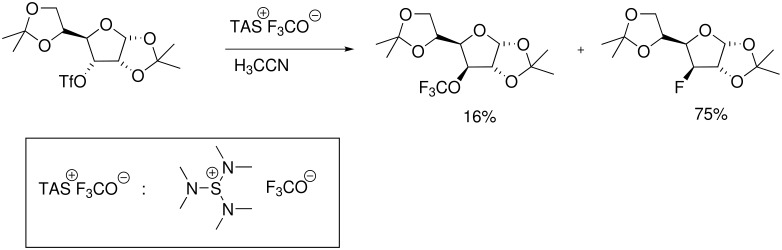
TAS OCF_3_ as a nucleophilic OCF_3_-transfer agent.

The introduction of the trifluoromethoxy substituent into carbohydrates was realized using *tris*(dimethylamino)sulfonium trifluoromethoxide (TASOCF_3_) as OCF_3_-transfer reagent [39]. This compound can be prepared by reaction of carbonyl fluoride with *tris*(dimethylamino)sulfonium difluorotrimethylsilicate in anhydrous THF at −75 °C (Scheme 9) [40]. The trifluoromethoxide anion is a relatively poor nucleophile. However, when reacted with primary triflate esters of carbohydrates, the anion displaced the triflate under mild conditions.

However, although aromatic trifluoromethyl ethers are well known and have many applications in pharmaceutical and agricultural domains, aliphatic trifluoromethyl ethers are still rare and difficult to make [41]. Methyl (trifluoromethoxy)acetate for example has been prepared [42] using the carbonyl fluoride/sulfur tetrafluoride method cited above [36]. Recent advances in the fluorodesulfurization reaction [24,41,43–45] enabled the preparation of some aliphatic trifluoromethyl ethers under mild conditions.

### Properties

**Figure 2:**
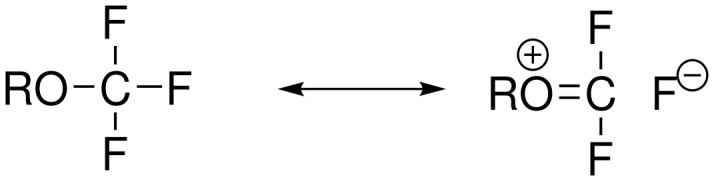
Mesomeric structures of the OCF_3_-group.

What makes the introduction of OCF_3_ into pharmaceutically relevant compounds particularly intriguing is their unique electron distribution. The geminal combination of an alkoxy or aryloxy group with a fluorine atom offers the possibility of bonding/non-bonding resonance which can be formally expressed by the superposition of a covalent and an ionic limiting structure (Figure 2).

#### Structure

The anomeric effect reveals itself by a lengthening of the acceptor bond and a shortening of the donor bond. The differences are nevertheless small at least as far as the carbon-fluorine bond is concerned which in general is elongated by just 0.02 Å. In contrast, anomerically active carbon-oxygen bonds may shrink by almost 0.1 Å. The stable tris(dimethylamino)sulfonium (TAS) trifluoromethoxide (see Scheme 9 for structure) [40] represents an extreme case. The three carbon-fluorine bonds are stretched by approximately 0.07 Å and the carbon-oxygen bond contracted by 0.09 Å relative to trifluoromethanol [40] and by 0.21 Å relative to methanol [46].

**Figure 3:**
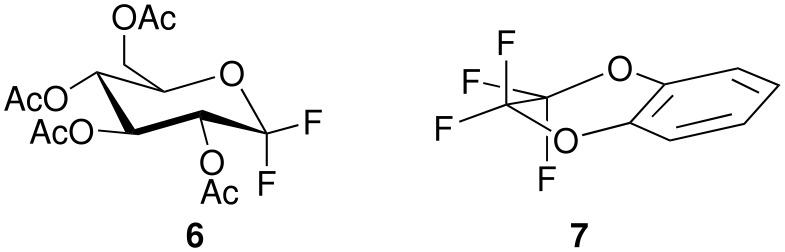
Structures of **6** and **7**.

The geminally 1,1-difluorinated 2,3,4,6-tetra-*O*-acetyl-1-deoxy-D-glucopyranose (**6**, Figure 3) [47] exhibits unequivocally non-identical C-F bond lengths, according to crystallography. The difference of 1.5 hundredth of an Å falls in the expected range.

2,2,3,3-Tetrafluoro-2,3-dihydro-1,4-benzodioxine (**7**, Figure 3) which occupies a half-chair conformation, shows clearly differences between quasi-axial and quasi-equatorial fluorine atoms. The average bond lengths of 1.355 and 1.330 Å differ significantly [48].

The effect of replacing a methyl by a trifluoromethyl moiety on bond length is dependent upon the electronegativity of the atom to which the substituent is attached [49] and reflects the "anomeric effect" shown above [50]. The lengthening of the acceptor bond and the shortening of the donor bond are small, as far as the carbon fluorine bond are concerned. However the carbon-oxygen bond may decrease by almost one tenth of an Å (Table 6).

**Table 6:** Effect of substituting a trifluoromethyl group for methyl on different heteroatoms.

Atom/group Y-CX_3_ (X = H, F)	Allred-Rochow Electronegativity	C-Y bond length in Å		Δr = r(CF_3_)–r(CH_3_) in Å
		X = H	X = F	

P-(CX_3_)	2.06	1.844	1.904	+0.060
H-(CX_3_)	2.20	1.099	1.102	+0.003
I-(CX_3_)	2.21	2.139	2.138	−0.001
S-(CX_3_)	2.44	1.805	1.819	+0.014
Se-(CX_3_)	2.48	1.945	1.980	+0.035
Br-(CX_3_)	2.74	1.939	1.923	−0.016
Cl-(CX_3_)	2.83	1.781	1.752	−0.029
N-(CX_3_)	3.07	1.458	1.426	−0.032
O-(CX_3_)	3.50	1.416	1.369	−0.047
F-(CX_3_)	4.10	1.385	1.319	−0.066

**Figure 4:**
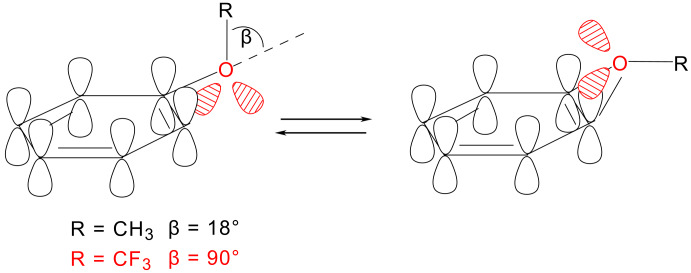
Conformational preference of the trifluoromethoxy group on aryl rings.

A fluorine substituent can lead to a change in the preferred molecular conformation. For example, methoxybenzenes without *ortho* substituents favor a planar conformation. However, Roche researchers by searching trifluoromethoxybenzenes without *ortho* substituents in the Cambridge Structural Database, found that none of the entries has the –OCF_3_ group in the plane of the phenyl ring (Figure 4). From six compounds, five entries have a dihedral angle C-C-O-C of around 90° and one compound showed a skew conformation (dihedral angle C=C/OCF_3_ : 36°) [51].

#### Lipophilicity

On the basis of its electronic properties, which are close to those of a chlorine or a fluorine atom [52], the trifluoromethoxy group has been referred to as a super- [53] or a pseudo-halogen [54]. The advantage of incorporating a trifluoromethoxy group into a molecule can be described in terms of its properties. The trifluoromethoxy group is both more electron withdrawing and lipophilic than its methoxy analogue.

The fluorinated carbon adjacent to an oxygen atom increases lipophilicity as shown by the high value of the OCF_3_ hydrophobic substituent parameter. While both trifluoromethyl and trifluoromethoxy substituents invariably boost the lipophilicity (Table 7), single fluorine atoms may alter this parameter in either direction. If the halogen occupies a vicinal or homovicinal position with respect to a hydroxy, alkoxy or carbonyl oxygen atom, it enhances the solvation energy in water more than in organic solvents (such as 1-octanol or chloroform) and hence lowers the lipophilicity [51]. It appears that the OCF_3_ substituent is far more lipophilic (π = +1.04) than the halogens and lies between a CF_3_ (π = +0.88) and a SCF_3_ (π = +1.44) group. It may thus replace advantageously a fluorine atom (π = +0.14) in most molecules with the benefit of increased lipid solubility.

**Table 7:** Electronegativities and Hydrophobic Parameters for various substituents.

Atom/group	Pauling Electronegativity	Hydrophobicity π [55–56]

H	2.1	0.00
F	4.0	0.14
Cl	3.0	0.71
Br	2.8	0.86
I	2.5	1.12
CH_3_	2.3	0.56
C(CH_3_)_3_	2.3	1.98
CF_3_	3.5	0.88
OCH_3_	2.7	−0.02
OCF_3_	3.7	1.04
SCF_3_	–	1.44
C_6_H_5_	–	1.96
SF_5_	–	1.23

#### Acidity of trifluoromethyl ethers

As described previously, the trifluoromethoxy group is at the same time a strong electron-withdrawing substituent due to the three fluorine atoms and a π-donating substituent due to the oxygen lone pairs. Yagupol'skii [57–58] and Sheppard [53,59] provided detailed data on the p*K*_a_-values of benzoic acids and phenols which reveal that the trifluoromethoxy group is a moderately electron-withdrawing moiety which resembles a chlorine atom. The p*K*_a_ values are lowered by the trifluoromethoxy group by 0.5 – 1.0 units [60–62].

### Reactivity

The OCF_3_ group is thermally and chemically resistant to attack by acids, bases, organometallic reagents and oxidizing/reducing agents [23,36]. When substituted on an aromatic ring, the trifluoromethoxy group exhibits similar electron withdrawing behavior to the alkoxy group but also acts to deactivate the aromatic ring system [53].

### Electrophilic Aromatic Substitution

Trifluoromethoxybenzene, for example, undergoes nitration considerably (up to 5 times) more slowly than benzene. The electrophilic substitution occurs selectively at the *ortho* and *para* position. This means the inductive electron-withdrawing effect compromises the attack of the electrophile, but is counterbalanced, to some extent, by the capacity of the ether oxygen to act through resonance as an electron donor. This antagonistic behavior is well known for chloro and bromo substituents. The trifluoromethoxy substituent has a pronounced preference for the *para *substitution. Unless the *para* position is occupied, *ortho* isomers are formed only in small amounts (≤ 10%) without any trace amounts of the *meta* isomers [52,63–64].

**Scheme 10:**
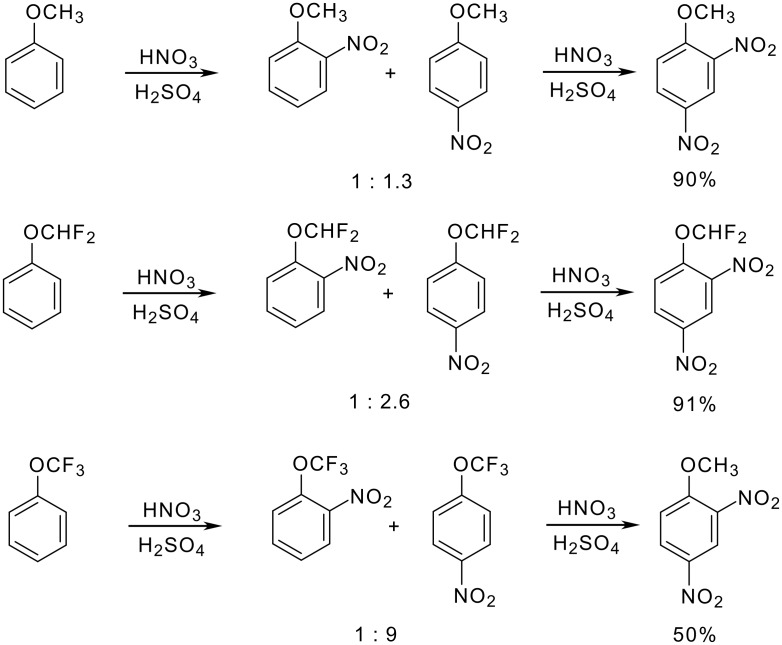
Nitration of trifluoromethoxy benzene.

When nitration is carried out under standard conditions, the *ortho/para* ratio changes with the number of fluorine atoms as depicted in Scheme 10 [52,65–66]. At temperatures in the range of 25 – 50 °C, double nitration can be achieved. The resulting 2,4-dinitrophenyl ethers are isolated in moderate to excellent yield [29,66–67].

**Scheme 11:**
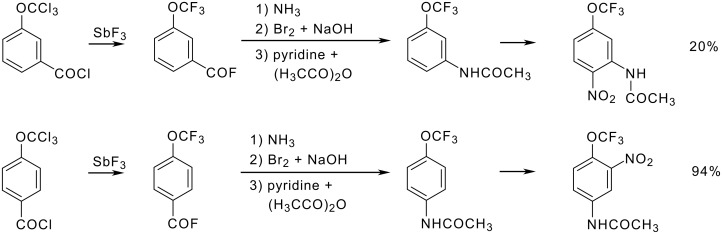
Synthesis and Nitration of *N*-Acetyl-(trifluoromethoxy)anilines.

The *para*-directing effect of a trifluoromethoxy group surpasses even that of an amide function. *N*-Acetyl-3-(trifluoromethoxy)aniline is nitrated mainly at the 6-position and to a minor extent (10%) at the 4-position (Scheme 11) [19]. *N*-Acetyl-4-(trifluoromethoxy)aniline reacts at the 3-position (again *meta* with respect to the nitrogen function and *ortho* to the trifluoromethoxy group!).

The pronounced preference for *para* substitution of (trifluoromethoxy)benzene [52,63–64] holds for most electrophilic aromatic substitutions, in particular sulfonation [64], bromination [52], chloromethylation [68] and acylation [52,64]. Attack at the *meta* position has so far been observed only with the isopropylation and ethylation of (trifluoromethoxy)benzene (to the extent of 9 and 31%, respectively) [52].

### Organometallic Reactions

Some very versatile methodology functionalizing trifluoromethoxy substituted aromatics is based on the synthesis-oriented organometallic chemistry. The metal is introduced into a substrate in general by either one of two favorite methods, the permutational interconversion of halogen against metal or hydrogen against metal and subsequently replaced by an electrophile [69–70].

**Scheme 12:**
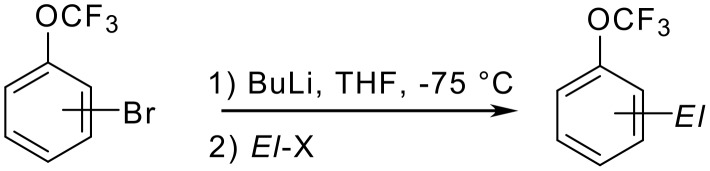
Bromine/lithium exchange of bromo(trifluoromethoxy)benzenes.

The three isomeric bromo(trifluoromethoxy)benzenes react easily with butyllithium in diethyl ether at −75 °C to generate the corresponding aryllithium (Scheme 12) species which can be trapped by a variety of electrophiles furnishing a diversity of new products (Table 8) [71–72].

**Table 8:** Reaction of 2-, 3- and 4-(trifluoromethoxy)phenyllithiums and electrophiles.

*El*	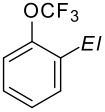	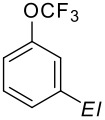	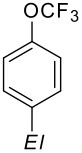

B(OH)_2_	89%	72%	84%
OH	88%		
Br	71%		
I	81%	74%	86%
CH_3_	52%	77%	73%
CH_2_CH_2_OH	<5%	78%	70%
CHO	93%	90%	95%
COCOOC_2_H_5_	87%	63%	61%
COCH_2_COOC_2_H_5_	52%	32%	26%
COOH	80%	85%	95%
CN	49%		21%

**Scheme 13:**
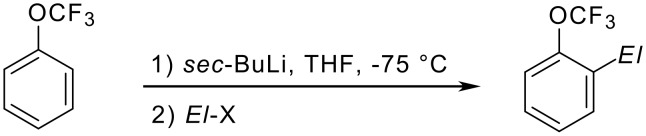
Metalation of (trifluoromethoxy)benzene.

Trifluoromethoxybenzene reacts with *sec*-butyllithium in the presence of *N,N,N',N'-*tetramethylethylenediamine ("TMEDA") smoothly under hydrogen/metal permutation ("metalation") as shown in Scheme 13 [72].

**Scheme 14:**
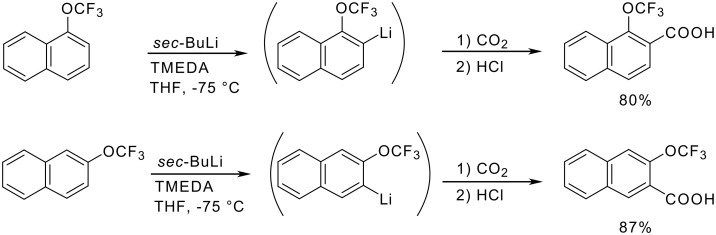
Metalation of (trifluoromethoxy)naphthalenes.

4-Trifluoromethoxybiphenyl can be metalated using the Schlosser superbase LIC-KOR made by combining butyllithium (LIC) with potassium *tert*-butoxide (KOR) in tetrahydrofuran at −100 °C. Upon trapping with molecular iodine, 3-iodo-4-trifluoromethoxybiphenyl was isolated in 90% yield [73]. Under the same conditions as employed with trifluoromethoxybenzene, 1- and 2-trifluoromethoxynaphthalene undergo selective lithiation at the 2- and 3-position, respectively (Scheme 14) [74].

**Scheme 15:**
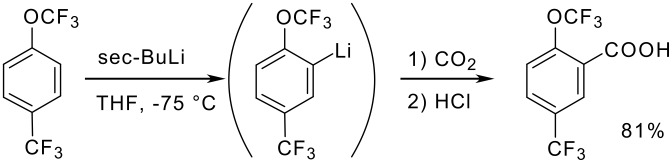
Competition between -CF_3_- and -OCF_3_ in Metalation reactions.

Both the trifluoromethyl and the trifluoromethoxy group are strongly electron-withdrawing groups and both have a far-reaching activating effect [74]. In an intramolecular competition on 1-trifluoromethoxy-4-(trifluoromethyl)benzene it has been shown, that lithiation next to the OCF_3_ substituent is favoured, probably due to steric reasons. In fact, 1-trifluoromethoxy-4-(trifluoromethyl)benzene (Scheme 15) affords 2-trifluoromethoxy-5-(trifluoromethyl)benzoic acid after lithiation and carboxylation [75].

**Scheme 16:**
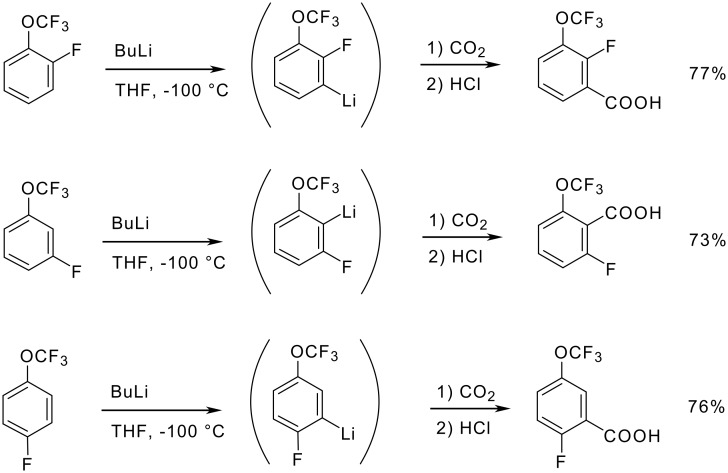
Competition between -F- and -OCF_3_ in Metalation reactions.

When the OCF_3_ substituent is in competition with fluorine, as in fluoro(trifluoromethoxy)benzenes, the fluorine-adjacent positions are always metalated (Scheme 16) [75].

**Scheme 17:**
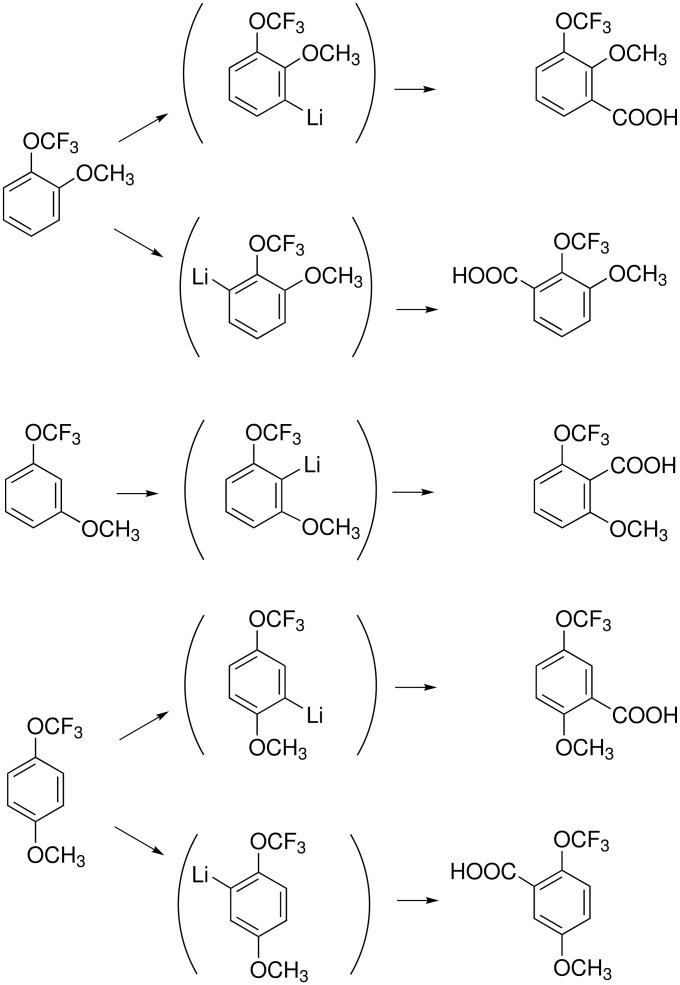
Metalation of trifluoromethoxyanisoles.

The OCF_3_ group reveals a powerful π-polarization as it acidifies not only the *ortho* but also the *meta* and *para* positions strongly. Therefore, metalation of 2- and 4-(trifluoromethoxy)anisole occurs preferentially or exclusively at the methoxy-neighboring position. However, proton abstraction at the trifluoromethoxy-adjacent sites becomes dominant when *sec*-butyllithium in the presence of *N*,*N*,*N*',*N*'',*N*''-pentamethyldiethylenetriamine (PMDTA) is employed. 3-Trifluoromethoxyanisole undergoes deprotonation always at the doubly activated 2-position (Scheme 17). The trifluoromethoxy group enhances the kinetic acidity of anisole by a factor of 3 if in the *ortho* position, 300 if in the *para* position and almost 2000 if in the *meta* position [76].

**Figure 5:**
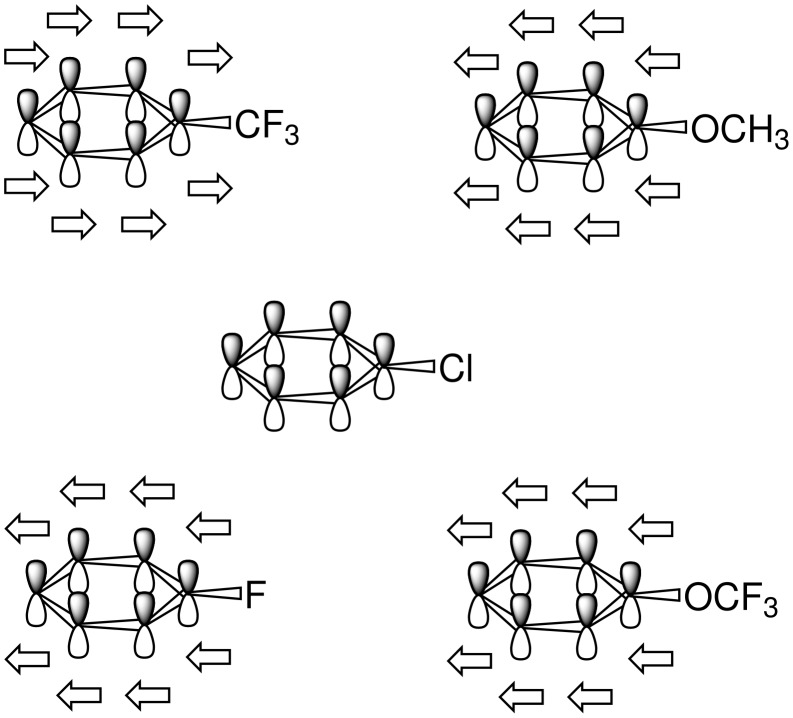
Direction of π-polarization depending on the substituent as described by Schlosser *et al.* [57].

The long-range effect of the trifluoromethoxy group was rationalized by Schlosser *et al.* by a synergy between two kinds of electronic perturbations. The electronegativity of nitrogen, oxygen, or a halogen atom pulls electrons in all σ-bonds towards the heteroelement. This σ-polarization diminishes with the distance. On the other hand, the substituent affects the π-electron cloud by attracting the whole sextet as one toward itself if it is both tetravalent and electrondeficient, e.g. trifluoromethyl or trimethylammonio groups. Alternatively, the π-cloud will remain, as in chlorobenzene, or even be pushed away from lone-pair carrying substituents (with progressively increasing strength from fluorine to alkoxy to dialkylamino). In this way, π-electron density can accumulate at the *meta* and *para* positions, where it counterbalances the σ-polarization. The trifluoromethoxy group has a slightly smaller σ-inductive effect than fluorine or a trifluoromethyl substituent. Its π-donating capacity is inferior to the one of the methoxy group, and even inferior to that of a fluorine atom. As a result, these two effects confer its electronic individuality to the trifluoromethoxy group. While acidifying *ortho*-positions only moderately, its anion-stabilizing effect is important at *meta-* and *para-*positions (Figure 5) [76].

**Scheme 18:**
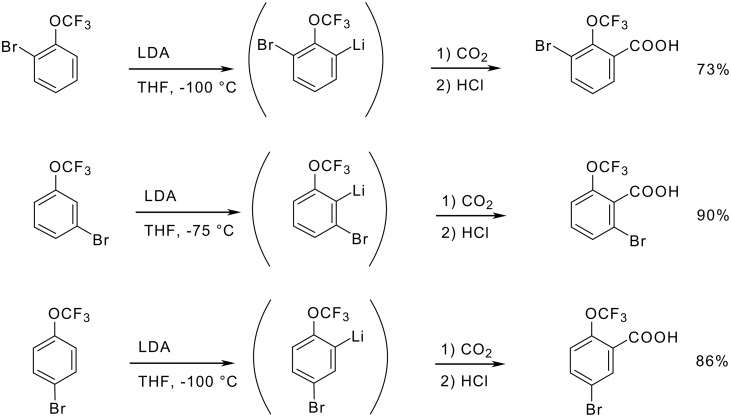
Metalation of Bromo(trifluoromethoxy)benzenes.

By contrast, bromo(trifluoromethoxy)benzenes are metalated at −100 °C by bases such as LDA at a position next to the oxygen substituent (Scheme 18) [74].

**Scheme 19:**
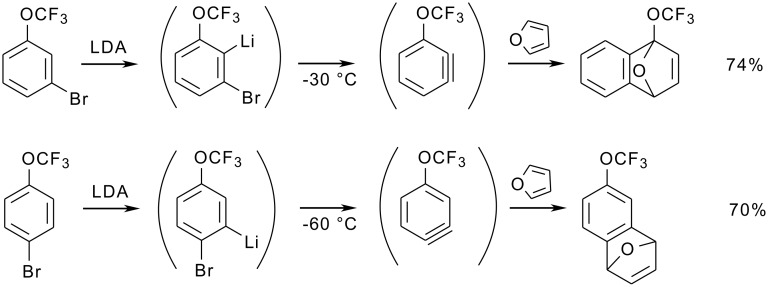
Aryne formation from bromo(trifluoromethoxy)phenyllithiums and subsequent Diels-Alder cycloaddition with furan.

At temperatures above −75 °C, lithium bromide elimination generates didehydro(trifluoromethoxy)benzenes ("arynes"). These short-lived species can be trapped with furan to form the corresponding Diels-Alder cycloadducts (Scheme 19) [74].

**Scheme 20:**
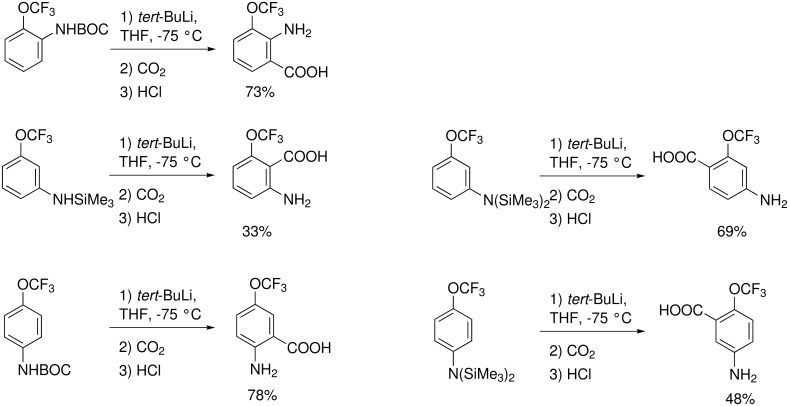
Metalation of (trifluoromethoxy)anilines.

Trifluoromethoxy substituted anilines require protection of the amino function. The BOC-protected *ortho* and *para* isomer gives the 3- and 4-(trifluoromethoxy)anthranilic acid after metalation with *tert*-butyllithium, followed by carboxylation (Scheme 20) [71]. When the amino function is protected instead of a BOC group by a silyl group, 3-trifluoromethoxy-*N*-(trimethylsilyl)aniline is metalated in position 2. However, 3- and 4-trifluoromethoxy-*N*,*N*-bis(trimethylsilyl)aniline are metalated at the oxygen-adjacent position [71].

## Conclusion

In the life science field, single fluorine atoms, trifluoromethyl or trifluoromethoxy groups are used to tailor p*K*_a_ values, to facilitate cell membrane penetration and to increase the metabolic stability of compounds. These features of fluorine contribute to the critical "bioavailability" of therapeutically active compounds. The growing interest and utility of the trifluoromethoxy-substitutent in drugs and agrochemical products, presents challenging synthetic strategies which are increasing being tackled in industrial and academic research programmes.
